# Ultra-Low-Dose Pre-Metallation Strategy Served for Commercial Metal-Ion Capacitors

**DOI:** 10.1007/s40820-022-00792-x

**Published:** 2022-01-29

**Authors:** Zirui Song, Guiyu Zhang, Xinglan Deng, Kangyu Zou, Xuhuan Xiao, Roya Momen, Abouzar Massoudi, Wentao Deng, Jiugang Hu, Hongshuai Hou, Guoqiang Zou, Xiaobo Ji

**Affiliations:** 1 College of Chemistry and Chemical Engineering, Central South University, Changsha, 410083 People’s Republic of China; 2 Department of Semiconductors Materials and Energy Research Center, P.O. Box 14155/4777, Tehran, Iran; 3 College of Material Science and Engineering, Zhengzhou University, Zhengzhou, 450001 People’s Republic of China

**Keywords:** Coupled interface, Pre-metallation, Metal oxalate, Decomposition potential

## Abstract

**Highlights:**

- Interfacial bonding strategy has been successfully applied to address the high overpotential issue of sacrificial additives, which reduced the decompositon potential of Na_2_C_2_O_4_ from 4.50 to 3.95 V.
- Ultra-low-dose technique assisted commercial sodium ion capacitor (AC//HC) could deliver a remarkable energy density of 118.2 Wh kg^−1^ as well as excellent cycle stability.
- In-depth decomposition mechanism of sacrificial compound and the relative influence after pre-metallation were revealed by advanced in situ and ex situ characterization approaches.

**Abstract:**

Sacrificial pre-metallation strategy could compensate for the irreversible consumption of metal ions and reduce the potential of anode, thereby elevating the cycle performance as well as open-circuit voltage for full metal ion capacitors (MICs). However, suffered from massive-dosage abuse, exorbitant decomposition potential, and side effects of decomposition residue, the wide application of sacrificial approach was restricted. Herein, assisted with density functional theory calculations, strongly coupled interface (M–O–C, M = Li/Na/K) and electron donating group have been put forward to regulate the band gap and highest occupied molecular orbital level of metal oxalate (M_2_C_2_O_4_), reducing polarization phenomenon and Gibbs free energy required for decomposition, which eventually decrease the practical decomposition potential from 4.50 to 3.95 V. Remarkably, full sodium ion capacitors constituted of commercial materials (activated carbon//hard carbon) could deliver a prominent energy density of 118.2 Wh kg^−1^ as well as excellent cycle stability under an ultra-low dosage pre-sodiation reagent of 15–30 wt% (far less than currently 100 wt%). Noteworthily, decomposition mechanism of sacrificial compound and the relative influence on the system of MICs after pre-metallation were initially revealed by in situ differential electrochemical mass spectrometry, offering in-depth insights for comprehending the function of cathode additives. In addition, this breakthrough has been successfully utilized in high performance lithium/potassium ion capacitors with Li_2_C_2_O_4_/K_2_C_2_O_4_ as pre-metallation reagent, which will convincingly promote the commercialization of MICs.

**Supplementary Information:**

The online version contains supplementary material available at 10.1007/s40820-022-00792-x.

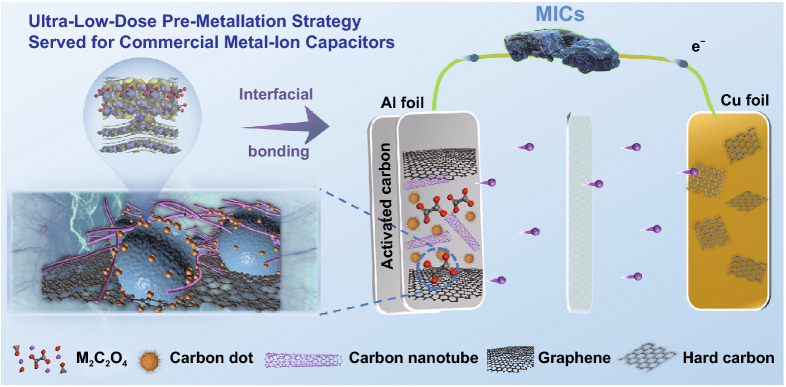

## Introduction

Metal ion capacitors (MICs), as the combination of metal ion batteries (large energy density) and supercapacitors (high power density), were built for next-generation energy storage systems [1–5]. Nevertheless, the deficiency of non-metal content nature in activated carbon associated with physically capacitive behavior (as cathode), seriously restricts the development of MICs due to the side reaction of metal ion consumption caused by the formation of solid electrolyte interphase (SEI) in the anode part [6–10]. Hence, pre-metallation was proposed as an indispensable strategy of supplying extra metal sources to ensure adequate metal ion reserved in the system of MICs, which could guarantee the stability of electrolyte, thus enhancing the cycle performance of MICs [11–13]. More importantly, released metal species would be transported to the anodes during charging process, then engendered the result of decreased potential of anodes, thereby elevating the voltage window as well as the energy density of full MICs [14–16].

Up to now, pre-metallation strategies can be divided in to several categories: operation with metal, usage of metal alternatives and introduction of additives [17–21]. Generally, the metal-operating methods contain the electrochemical and direct contact tactics, both can be carried out with simple procedures. Nevertheless, rigorous inert environment (Ar atmosphere, H_2_O level < 0.1 ppm and O_2_ level < 0.1 ppm) is required, which seriously restricts the corresponding scale-up application [22, 23]. As for the alternative method based on chemical metalation (e.g., the Li-biphenyl-tetrahydrofuran and Na-naphthalene-tetrahydrofuran solution), volatile, and flammable solvents are normally employed to dissolve the metal-based compound, which potentially induce severe safety accident [24–26]. In addition, such pre-metallation reagents are unable to be utilized during the slurry process directly, as it would interact with the common polar solvent (i.e., N-methyl pyrrolidone), which means the defect of intricate production process as well as the latent rising cost. Noteworthily, sacrificial cathode additives could be straight handled with active electrode material to achieve degree-controlled in situ pre-metallation, which exhibits excellent compatibility with existing fabrication process, shedding light to large-scale manufactures [27]. Significantly, three basic principles should be followed to select the desired additive: (1) High irreversible capacity under suitable voltage window. (2) Chemical stability and environmentally friendliness. (3) No residue after activation. Sodium ion capacitors (SICs) possess the superiority of abundant reserve in the crust and relatively high energy density compared with lithium ion capacitors (LICs) and potassium ion capacitors (KICs) [28], in which numerous sacrificial compounds have been successfully applied (e.g., Na_2_S [29], NaNH_2_ [30], Na_2_C_4_O_4_ [31, 32], Na_2_C_6_O_6_ [33], Na_2_C_6_H_2_O_6_). However, dead mass phenomenon, safety hazard or relatively low irreversible capacity limits their further development. Among all the cathode additives, vital characteristics including decomposition product, theoretical capacity, dosage, cost, and purity are summarized as evaluation criteria (Table S1), it is discovered that sodium oxalate holds the possibility to be identified as perfect candidate as it demonstrates prominent comprehensive capabilities. Most importantly, extremely low-cost advantage and air-stable ability make it feasible to be adopted in industrial application. Nevertheless, the high decomposition potential of Na_2_C_2_O_4_ seriously hinders the irreversible output capacity, accordingly causing the dosage-abuse issue. Moreover, exorbitant activation potential may lead to the break-down of electrolyte on the cathode side and potential unsafe reactions, resulting in poor electrochemical performance, thereby impairing the practical application of SICs [34, 35].

Herein, as predicted by the density functional theory (DFT) calculations, Gibbs free energy of irreversible oxidation process of sacrificial additive can be decreased accompanied with rising highest occupied molecular orbital (HOMO) level when utilizes the electron-donating effect by introducing methylene to alkyl chain to impair the interaction between carboxylate and Na, thus obtaining reduced decomposition potential. Furthermore, strongly coupled interface (M–O–C) has been introduced to regulate the band gap of metal oxalate, largely reducing polarization phenomenon, which effectively decrease the practical decomposition potential. Significantly, interfacial pseudo-bonding Na–O–C between Na_2_C_2_O_4_ and 3D conductive network could facilitate the charge transfer ability. In addition, reduced particle size can curtail the long transmission path of Na^+^, then boosts the ion conductivity. Consequently, the decomposition potential of sodium oxalate has been diminished to satisfied extent (3.95 V). Notably, compared with the dosage situation of sacrificial additives in the current research work, ultra-low addition of ameliorated sodium oxalate compound in commercial SICs can achieve extraordinary energy density (118.2 Wh kg^−1^) and preeminent cycle performance with the assistance of original double coating tactic. Besides, the corresponding scale-up feasibility has been proven by pouch-type capacitors with a practicable energy density of 40.5 Wh kg^−1^ after pre-sodiation. In-depth decomposition mechanism and relevant influence on the whole system of sacrificial additives have been systematically investigated by in situ differential electrochemical mass spectrometry and ex situ X-ray photoelectron spectrometry. Finally, the versatility and feasibility of Li_2_C_2_O_4_/K_2_C_2_O_4_ as additives have been successfully verified in the configuration of LICs and KICs, respectively, dedicating to promote the industrialization of MICs.

## Experimental Section

### Materials and Methods

#### Preparation of Metal Oxalate Cathode Electrodes

The mixed slurry consisted of 50 wt% active material (M_2_C_2_O_4_), 10 wt% binder polyvinylidene fluoride (PVDF), and 40 wt% conductive additives dissolved in N-methyl pyrrolidinone (NMP), which were further coated on aluminum foil and the as-prepared cathode electrodes were dried under vacuum at 120 °C for 12 h. Graphite, graphene and carbon nanotube (CNT) were utilized as mono-conductive additives respectively in the electrochemical oxidation experiments. Three dimension (3D) conductive network was performed by introducing carbon dots, MCNT and graphene during the process of slurry. Size-reduced Na_2_C_2_O_4_ with 3D network (NCO-S-3D) was generated by high-energy ball milling for 12 h at a rotating speed of 1200 r min^−1^ with 3D conductive network to obtain the final product.

#### Preparation of Composite Cathode Electrodes

Traditional slurry method: The composite cathode electrodes were prepared by mixing 71.43 wt.% activated carbon (AC), 8.93 wt% conductive carbon (Super P), 8.93 wt% PVDF and 10.71 wt% Na_2_C_2_O_4_ with a mass ratio of 8:1:1:1.2. While the mass of additive is 15 wt% of AC. Double coating method: Firstly, the AC electrodes were composed of AC (80 wt.%), conductive carbon (10 wt%) and PVDF (10 wt%), which were dried under vacuum at 120 °C for 12 h. Then individual metal oxalate slurry was coated on the top of the AC electrodes, followed by vacuum drying at 120 °C overnight. The various mass ratio of metal oxalate/AC can be precisely controlled by the thickness of scraper.

#### Preparation of Anode Electrodes

Commercial anatase TiO_2_ was chosen as anodes which were consisted of 70 wt% active material, 15 wt% conductive carbon (Super P) and 15 wt% binder carboxymethyl cellulose. The components were mixed with deionized water to obtain a homogeneous ink. Then the ink was coated on a Cu foil and the as-prepared anode electrodes were dried under vacuum at 80 °C for 12 h.

### Materials Characterization

X-ray diffraction (XRD) patterns were recorded by X-ray diffractometer (Rigaku, Japan) with a Cu-Kα radiation of 0.15418 nm. The surface morphologies of the prepared samples were analyzed by the scanning electron microscopy (SEM, Hitachi S-4800) and transmission electron microscopy (TEM, JEOL JEM 2100F). The detailed compositions were determined by using an X-ray photoelectron spectroscopy analyzer (XPS) (VG Multi Lab 2000 system) and Fourier transform infrared spectrometer (FTIR) (Bruker Equinox 55 spectrometer). Gas evolution was detected by differential electrochemical mass spectrometry (DEMS) (Linglu Instruments Co., Lt).

### Electrochemical Measurements

In order to investigate pre-metallation behaviors of various metal oxalates and the effect of conductive additives in electrochemical oxidation experiment, the metal oxalate cathodes were employed as the working electrodes in half-cells. Meanwhile, the corresponding alkali-metals (lithium, sodium or potassium) were utilized as the counter and reference electrodes. Furthermore, the electrolytes used for different electrochemical energy storage (EES) systems were 1 mol L^−1^ LiPF_6_ solution in ethylene carbonate (EC), ethyl methyl carbonate (EMC) and dimethyl carbonate (DMC) (1:1:1 in vol%) with 5 wt% fluoroethylene carbonate (FEC), 1 mol L^−1^ NaClO_4_ solution in EC, EMC and DMC (1:1:1 in vol.%) with 5 wt% FEC and 0.8 M KPF_6_ solution in EC:DMC (1:1 in vol%), respectively. A Whatman GF/C glass fiber membrane was utilized as the separator. All EES devices were assembled with respective anode/cathode in the Braun glovebox with high purity argon atmosphere and all related electrochemical measurements were performed at room temperature. To obtain the available MIC cells with the additives of metal oxalates, the electrochemical activation should be carried out to achieve in situ pre-metallation by cycling for one time under the current density of 10 mA g^−1^. Then the MICs were left at open-circuit potential (OCP) for 12 h to allow electrolyte penetration into the porosity of the electrodes. The mass loadings of anodes in MICs were 0.8 ~ 1.6 mg cm^−2^. Moreover, the mass loadings of composite cathodes (including AC/Li_2_C_2_O_4_, AC/Na_2_C_2_O_4_ and AC/K_2_C_2_O_4_) in MICs were about 5 mg cm^−2^.

CV curves with various scan rates were measured by a MULTI AUTOLAB M204 (MAC90086). Galvanostatic charge/discharge (GCD) surveys were recorded on an Arbin BT2000 instrument at diverse current densities within an appropriate voltage window. Electrochemical impedance spectroscopy (EIS) experiments were performed using the Chenhua electrochemical workstation (CHI660E, Chenhua Instrument Company, China) under open-circuit potential. Cycle-life tests were recorded on a Land CT2001A model battery system. Galvanostatic intermittent titration technique (GITT) measurement was programmed by supplying a constant current flux of 0.5 C for 30 min followed by an open-circuit stand for 8 h. The energy density and power density of SIC are determined according to the total mass of active material (including anode and cathode).

### DFT Calculations

DFT calculations were used to calculate the bonding energy of O–M moiety as well as Gibbs free energies. The electron interaction and the exchange functions were employed by the Perdew-Burke-Ernzerh functional (PBE) of the generalized gradient approximation (GGA) with semicore pseudopotentials (DSPPs) core treatment under polarized function (DNP) basis set. The convergence tolerance of energy, maximum displacement and the maximum force were set to 2 × 10^−5^ Hartree, 0.005 Å and 0.004 Hartree Å^−1^, respectively.

## Results and Discussions

### Theoretical Simulation

**Fig. 1:**
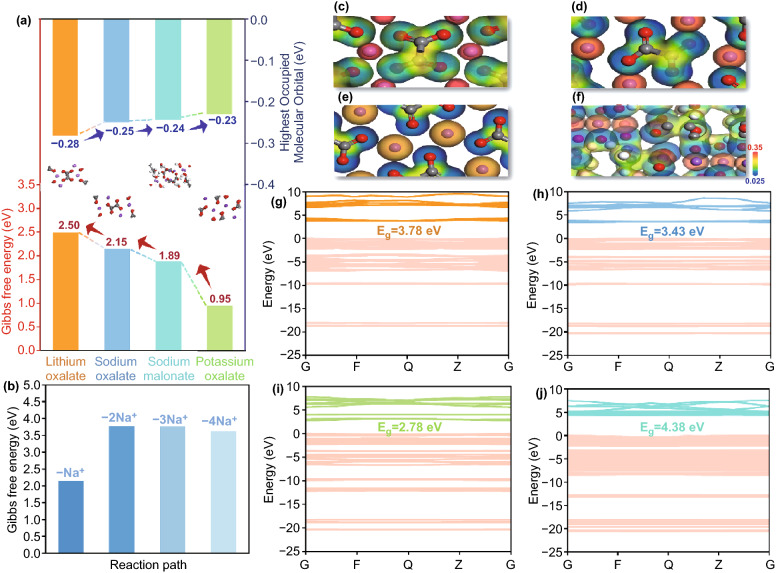
DFT calculations of M_2_C_2_O_4_ (M = Li/ Na/K) and sodium malonate. **a** Relative comparation of HOMO levels and Gibbs free energies (the insets are corresponding molecular structures). **b** Desodiation process of sodium oxalate. Electrostatic potential plots **c**-**f** and band structures **g**-**j** of Li_2_C_2_O_4_, Na_2_C_2_O_4_, Na_2_C_2_O_4_ and Na_2_H_2_C_3_O_4_

Note that sacrificial strategy applied in MICs relies on the irreversible decomposition of additive during the charging process to provide necessary sodium ions for anode part, then accomplishes the mission of pre-metallation. Therefore, modulating the decomposition procedure under suitable working voltage window holds the key to wield this methodology [36]. As shown in Fig. S1a, the CV measurements were carried out to illustrate the initial decomposition potential of Na_2_C_2_O_4_ with super p acting as conductive additive (marked as NCO-SP). It could be clearly observed that a sharp increase in the anodic current started from 4.50 V, which was the intersection of two auxiliary tangents, indicating the irreversible oxidation of Na_2_C_2_O_4._ Moreover, along with the curve, another break point occurred at 4.87 V, which was caused by the intense decomposition of electrolyte. To verify the irreversible capacity of NCO-SP, GCD experiments were conducted (Fig. S1b). It was shown that NCO-SP exhibited a specific capacity of 400 mAh g^−1^ (identical to the theoretical decomposition capacity) at the current density of 0.1 C (10 mA g^−1^) when charged to 4.56 V, which was consistent with the phenomenon discovered from CV curves. Furthermore, the second charge specific capacity of NCO-SP suffered a violent attenuation, which confirmed the capacity irreversibility of NCO-SP. However, such a high decomposition potential of sodium oxalate (4.50 V) could induce the latent break-down of electrolyte, sequentially incur the collapse of cycle performance. Hence, developing new strategies to reduce the activation potential is of prime importance to promote the wide application of Na_2_C_2_O_4._ Under the guidance of DFT calculations, the bonding energy of O-M bond can be obviously decreased triggered by the electron-donating effect of substituents like alkyl group. To be more specific, when adding a methylene group to the alkane chain, the electron cloud density of alkane chain gets enhanced as displayed in electrostatic potential plots (Fig. 1), resulting in the weakened interaction between carboxylate and sodium. As shown in Fig. 1a, the Gibbs free energy (ΔG) of desodiation process decreases from 2.15 to 1.89 eV accompanied with increased HOMO level, further bringing out reduced oxidation potential, which could achieve the preferable pre-sodiation purpose in SICs. In order to verify the DFT calculations, CV measurement of electrode composed of sodium malonate and super p was executed (Fig. S1c). It was illustrated that the decomposition potential of Na_2_H_2_C_3_O_4_-SP electrode was 4.30 V, 0.2 V lower than that of NCO-SP, which confirmed that the electron-donating effect could effectively diminish the binding strength of O-Na moiety, thus fulfilling the function of regulating the decomposition potential of sacrificial additives. In addition, as displayed in Fig. 1c–e, analogous interactions between metal ion (Li/Na/K) and O were clearly observed, comparable band gap (*E*_g_) of metal oxalate (Fig. 1g–i) further implied the feasibility to be utilized as pre-metallation reagents. The relevant validation experiment will be mentioned in the following section.

**Fig. 2:**
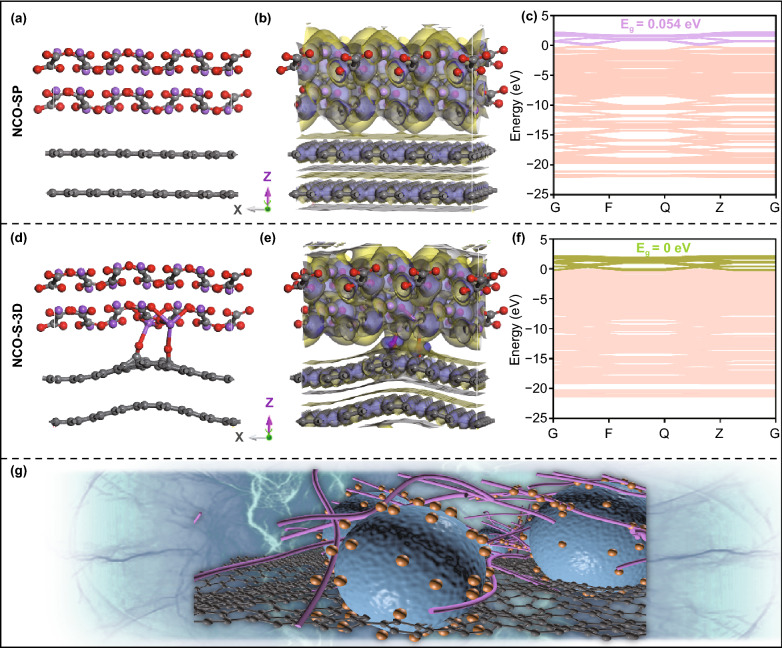
**a** Crystal structure, **b** charge density difference plot and **c** band structure of NCO-SP. **d** Crystal structure, **e** charge density difference plot and **f** band structure of NCO-S-3D. **g** Schematic illustration of interfacial bonding between Na_2_C_2_O_4_ and 3D conductive network (the orange ball, purple tube, black ball and blue ball represent carbon dot, carbon nanotube, graphene and sodium oxalate, respectively)

Although effect of electron-donating has been proved, additional CH_2_ provides no contribution to the extra sodium capacity, decreasing the theoretical capacity from 400 to 362 mA h g^−1^_._ Additionally, *E*_g_ of sodium malonate is wider than that of sodium oxalate as shown in Fig. 1j, which means that Na_2_H_2_C_3_O_4_ exhibits inferior conductivity. From the perspective of acquiring the most efficient sacrificial additive, cutting down redundant molecular weight to obtain the irreversible output capacity as large as possible is of necessity, sodium oxalate can still be identified as ideal research object in the field of sacrificial cathode additives. In addition, air-stability is considered as one of the prerequisites to realize the wide application of sacrificial additives, appearance observation and XRD results were found that no evident transformation of Na_2_C_2_O_4_ occurred during a long period of exposing in ambient condition (Fig. S2), indicating suitability of sodium oxalate for cosmically storage. Significantly, according to the theoretical calculations, ΔG of desodiating multiple Na^+^ process (Fig. 1b) from sodium oxalate unit cell are 3.78 ( − 2 Na^+^), 3.77 ( − 3 Na^+^), and 3.62 ( − 4 Na^+^) eV, respectively, which indicates that severe polarization plays the role of main culprit for high decomposition potential. Therefore, various attempt has been carried out to boost the conductivity of Na_2_C_2_O_4_ electrode, thereby addressing the exorbitant activation potential issue through mitigated overpotential hazard, the corresponding achievements are summarized in Table S2. Practically, graphene, graphite, and carbon nanotube (CNT) were separately utilized as mono-conductive additive instead of super p to mix with Na_2_C_2_O_4_ in half cells, manifesting certain reduction in decomposition voltage to 4.36, 4.38, and 4.40 V, respectively (Fig. S3a-c), based on the CV results. Moreover, three dimension (3D) conductive network composed of carbon dots (point), CNT (wire) and graphene (plane) was adopted as multi-conductive additive to slurry with Na_2_C_2_O_4._ With the merit of perfect combination of dots, wires, and planes, the stereo 3D conductive network filled the gaps between the sodium oxalate particles during the slurry process, which can maximize the electronic conductivity of the electrode, thus accelerating the speed of charge transfer in the bulk phase. As shown in Fig. S3d, the cathode decomposition potential was cut down to 4.30 V, proving the validity of slacking activation polarization. Noteworthily, inspired by the intriguing physicochemical properties of carbon dots (CDs) endowed by various surface functional groups (hydroxyl, carboxyl groups, etc.) [37], strategy of establishing interfacial bonding between 3D conductive network and sodium oxalate to exterminate overpotential hazard was put forward. In order to corroborate the relative practicability, DFT calculations were performed in advance to elucidate the deep understanding of interfacial coupling. From Fig. 2, it can be apparently observed that Oxygen-containing group of CDs linking with sodium ion to form Na–O–C pseudo-bonding. Importantly, electronic cloud density of interface was totally redistributed as the consequence of coupling effect. As illustrated in the charge density difference plots, interfacial bonding of NCO@CD (Fig. 2d–e) can identify the strong charge transportation between Na_2_C_2_O_4_ and carbon dot compared to the weak van der Waals force of two layers (Fig. 2a–b), which could accelerate the electrons migration from NCO to CDs, thus boosting the conductivity of sodium oxalate electrode. Moreover, first-principle calculations were arranged to predict the change of band structures caused by interfacial bonding (Fig. 2c, f). It was revealed that strong coupling effect can contribute to superior conductivity as the band gap completely disappeared from 0.054 eV (unbonded group), which implied that the Na–O–C bond can efficiently improve electronic conductivity of Na_2_C_2_O_4_. These results above indicated the availability of bonding strategy from the perspective of theoretical calculation, hence, tactic of high-energy balling milling sodium oxalate with 3D network (marked as NCO-S-3D) was arranged to establish interfacial bonds in order to confirm the concrete effectiveness in practical operations, the corresponding schematic illustration is seen in Fig. 2g.

**Fig. 3:**
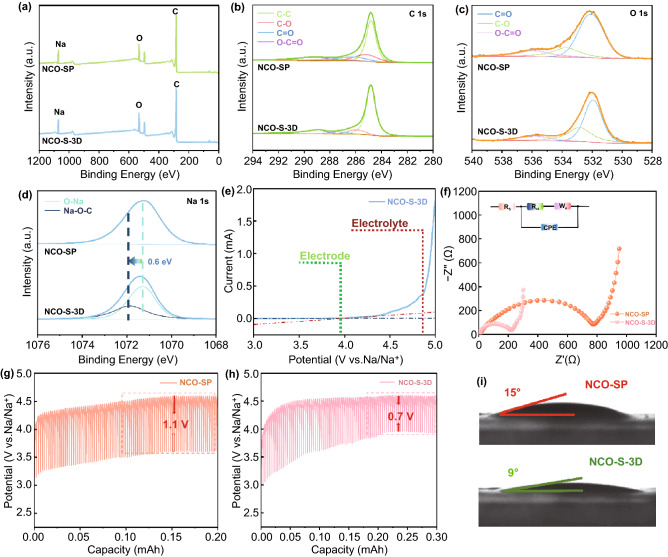
**a** XPS survey spectrum and **b** C 1 s, **c** O 1 s, **d** Na 1 s high-resolution XPS spectra of NCO-SP and NCO-S-3D. **e** CV curve of NCO-S-3D electrode at 0.25 mV s^−1^. **f** Comparison of EIS spectra of NCO-S-3D and NCO-SP. GITT curves of **g** NCO-SP and **h** NCO-S-3D. **i** Contact angle measurements of NCO-SP and NCO-S-3D

As depicted in Fig. 3a–d, XPS measurements were performed to identify the chemical environment of related elements and the coupling effect between Na_2_C_2_O_4_ and 3D network with NCO-SP group as comparison. The full survey scan spectrum (Fig. 3) revealed the existence of Na, C and O elements. Notably, compared to NCO-SP group, in addition to the peak related to Na–O (1071.27 eV), a distinctive peak shifted to higher binding energy (1071.87 eV), which was caused by the strong affinity of oxygen-containing groups in CDs, confirming the formation of Na–O-C bonds in NCO-S-3D heterostructures (Fig. 3d). Furthermore, detailed morphology of NCO-S-3D composite was depicted by transmission electron microscopy (TEM) in Fig. 5a–c. Obviously, CDs were uniformly deposited on whole configuration. High-resolution TEM (HRTEM) image exhibited in Fig. 5d revealed distinct lattice spacing of 0.26 nm, assigning to the (4 0 0) plane of Na_2_C_2_O_4._ Meanwhile, broad area of amorphous carbon was also detected, indicating that the CDs (marked with yellow circles) were tightly incorporated with sodium oxalate. Besides, the corresponding energy-dispersive spectroscopy (EDS) elemental mapping (Fig. S4) demonstrated the even distribution of Na, O, and C in NCO-S-3D compound, proving the successful introduction of CDs as well. Overall, these results demonstrated the intimate interaction between Na_2_C_2_O_4_ and CDs through a strong Na–O–C bonding, which was favorable for fast electron transfer, implying the great opportunity to diminish the activation potential of sodium oxalate compound. Remarkably, according to the CV results, decomposition potential of Na_2_C_2_O_4_ electrode was cut down to 3.95 V (Fig. 3e), which could efficiently prevent the electrolyte form oxidation.

In order to clarify the reduction mechanism of decomposition potential, techniques such as morphology observation, four-point probe measurement, electrochemical characteristics (EIS, GITT test) as well as contact angle measurement of NCO-S-3D and NCO-SP have been systematically analyzed. It was clearly revealed that ball milling method successfully decreased the particle size of Na_2_C_2_O_4_ from hundreds of microns to less than 1 micron (Fig. S5a-d). Reduced size can shorten the transmission path of sodium ions, consequently improves the ion conductivity thus ameliorating the concentration polarization. According to the results from four-point probe measurement, electronic conductivities were 6.25 × 10^–2^ and 8.62 × 10^–3^ S cm^−1^ for NCO-S-3D and NCO-SP, respectively, which verified that NCO-S-3D group possessed better electronic conductivity than NCO-SP. Moreover, distinctive difference was observed between the charge transfer impedance (*R*_ct_) of such two systems. Clearly, NCO-S-3D exhibited a much smaller *R*_ct_ than that of NCO-SP (Fig. 3f), indicating superior electron conductivity caused by interfacial bonding along with mitigated conductive agents, which was well consistent with the performance in four-point probe measurement. Besides, the polarization potential of NCO-S-3D demonstrated to be lower than NCO-SP (0.7 vs. 1.1 V) as revealed in GITT test (Fig. 3g–h), confirming the availability of above tactics. Additionally, the affinity of NCO (S-3D or SP) to electrolyte has been evaluated by contact angle measurements. Note that the contact angle (Fig. 3i) of NCO-S-3D electrode was found to be approximately 9°, which was smaller than that of NCO-SP (15°), illustrating the enhanced affinity triggered by the reduced particle size of Na_2_C_2_O_4_. It is worth mentioning that poor electrolyte infiltration effect would prolong the transmission path of Na^+^, which hinders the shuttle of sodium ions between electrodes [38]. More seriously, active materials which are not exposed to electrolyte cannot participate in the process of electrochemical reaction, meanwhile increasing the resistance of interface, thereby destroying the cycle life of SICs (more details in the following section). Overall, with the assist of enhanced conductivity as well as reduced particle size, interfacial bonded sodium oxalate eventually addressed the severe polarization issue with a satisfied activation potential of 3.95 V.

### Electrochemical Performance in Full Capacitors

**Fig. 4:**
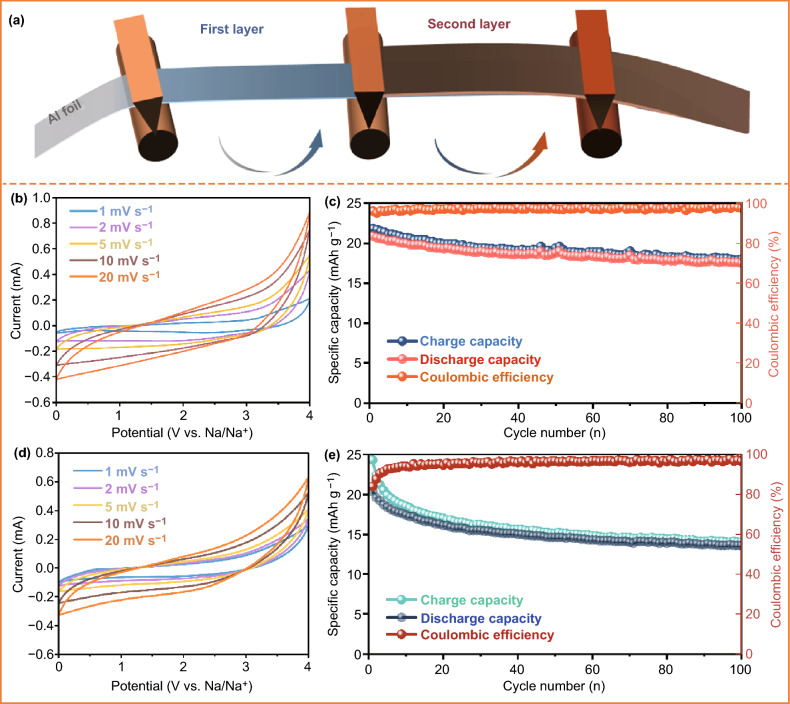
**a** Schematic illustration of double coating strategy. **b** CV curves of TiO_2_//AC-NCO-S-3D-15% at different scan rates after activation. **c** Cycling performance and CE of TiO_2_//AC-NCO-S-3D-15% under 1 C at 4–0 V after pre-sodiation. **d** CV curves of TiO_2_//AC-NCO-SP-15% at various scan rates after activation. **e** Cycling performance and CE of TiO_2_//AC-NCO-SP-15% under 1 C at 4–0 V after pre-sodiation

**Fig. 5:**
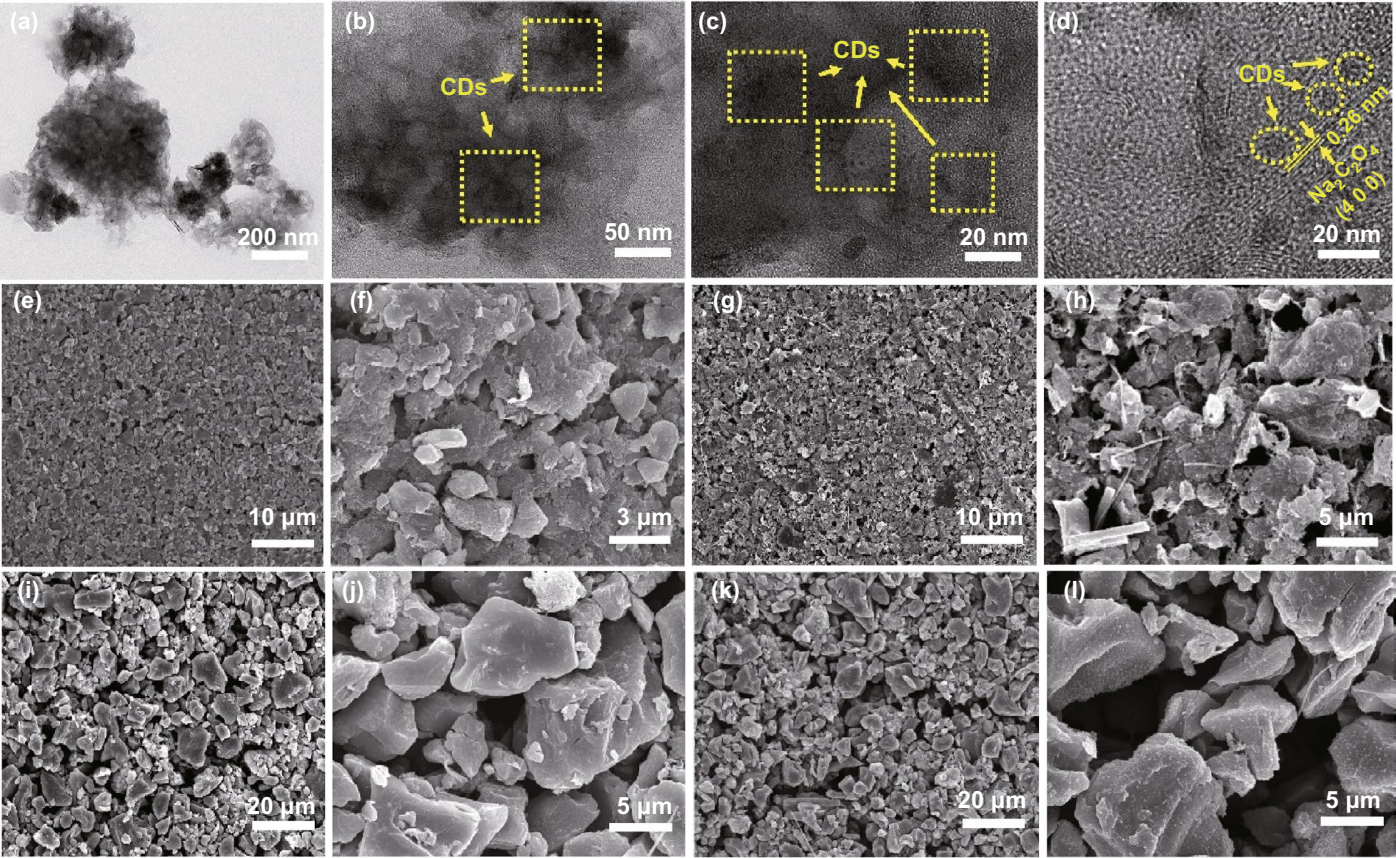
**a**-**c** TEM images of NCO-S-3D. **d** HRTEM image of NCO-S-3D. SEM images of decomposition characteristics of AC + NCO with double coating method before (**e, f**) and after (**g, h**) cycles. Surface images of AC + NCO with traditional slurry method before (**i, j**) and after cycles (**k, l**)

**Fig. 6:**
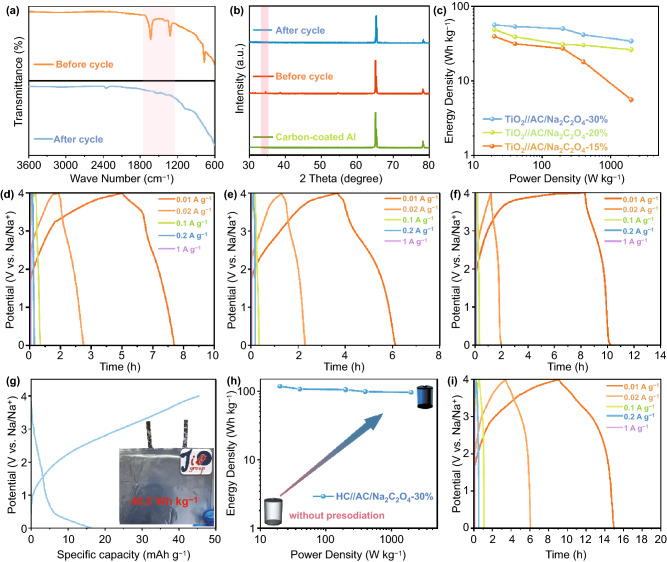
**a** XRD patterns of AC + NCO electrodes. **b** FTIR results of AC + NCO electrodes. **c** Ragone plots of TiO_2_//AC-Na_2_C_2_O_4_ with different dosage. Galvanostatic charge and discharge profiles at various current densities of **d** TiO_2_//AC-Na_2_C_2_O_4_-30%, **e** TiO_2_//AC-Na_2_C_2_O_4_-20%, and **f** TiO_2_//AC-Na_2_C_2_O_4_-15% SICs. **g** Charge and discharge profiles of pouch-type SIC at the current density of 0.1 C after activation. **h** The performance comparison of HC//AC without pre-sodiation SIC and HC//AC-Na_2_C_2_O_4_-30% SIC. **i** Galvanostatic charge and discharge profiles at various current densities of HC//AC-Na_2_C_2_O_4_-30% SIC

Noteworthily, it is discovered that fortified electrochemical performance of NCO-S-3D applied in full cells compared to the addition of NCO-SP exhibits the excellence of our ameliorated schemes. To verify the relative practical application in energy storage system, commercial materials were utilized as electrodes constructing full SICs (AC as cathode, TiO_2_ as anode) to investigate the cycle performance. According to the diverse sodium storage performance of electrodes (Fig. S6) and Eq. (1):

1 $$C_{{{\text{cathode}}}} *m_{{{\text{cathode}}}} = C_{{{\text{anode}}}} *C_{{{\text{anode}}}}$$

where C stands for the capacitance, m is the mass of electrode, respectively. It could be calculated that the suitable mass ration of TiO_2_ anode and AC cathode is about 1:5. Moreover, only 15 wt% of sacrificial additive (compare to the mass of AC) was required to compensate for the irreversible sodium loss in initial cycle due to the merit of massive irreversible capacity of Na_2_C_2_O_4_, thus holding the key to address the dosage abuse issue theoretically. Herein, 15 wt% various Na_2_C_2_O_4_ additive (S-3D or SP) were added to the system to measure the corresponding cycle stability. The open-circuit potential (OCP) of initiate assembled TiO_2_//AC-Na_2_C_2_O_4_ SICs were all around 0 V caused by the nonspontaneous pre-sodiation process. Thus, the full SICs cell were activated by GCD for one cycle at the current density of 0.1 C (10 mA g^−1^) within the potential range of 4.3–0 V. After the pre-sodiation treatment, full SICs manifested the distorted rectangular CV curves and not totally linear GCD slopes, respectively, illustrating that the collaborative coexistence of Faradaic and non-Faradaic charge storage mechanisms (Fig. 4) [39, 40]. It can be clearly observed that the TiO_2_//AC-NOC-S-3D-15% group delivered an impressive capacity retention of 83.7% after 100 cycles between 4 and 0 V at the current density of 1 C, which was 1.3 times higher than that of TiO_2_//AC-NCO-SP-15% group. Besides, initial coulombic efficiency of TiO_2_//AC-NCO-SP-15% full cell was only 83.9%, which was 12.1% lower than that of TiO_2_//AC-NCO-S-3D-15% full cell. This could be caused by the incomplete decomposition of NCO-SP during the activation procedure as it suffered from the severe polarization phenomenon. Noteworthily, advanced additive-coating technique as depicted in Fig. 4a was employed to boost the electrochemical performance of SICs instead of traditional slurry method. Based on the result in Fig. S7, the capacity retention of traditional slurry group was only 35.2% under the same circumstance. SEM images demonstrated relatively intact surface morphology of cathode after cycling for 100 cycles for the advanced additive-coating group (Fig. 5e–h), which should give credit to the double coating strategy as the Na_2_C_2_O_4_ decomposed on the surface of electrode, and the generated gas would be released without the structural collapse of AC. Interestingly, exfoliation of active material from current collector was not observed after dissembling full cells (Fig. S8), sequentially demonstrating the benefit of double coating strategy. Contrarily, plenty of holes and gaps were discovered in traditional set under the same condition due to the gas evolved in the bulk by the oxidation of Na_2_C_2_O_4_ (Fig. 5i–l), which caused the instability of the system thus inducing inferior cycle performance. In addition, to verify the complete decomposition of NCO-S-3D after activation process, the ex situ XRD and ex situ FTIR results were carried out. It was found that the reflection of electrode located at 34.4° indexed to lattice plane of (4 0 0) and two characteristic peaks of C=O stretching vibration of Na_2_C_2_O_4_ substance were absolutely disappeared after charging to 4.3 V (Fig. 6a–b), clearly revealing the first charging process enables sodium release from sodium oxalate [41]. Moreover, optical photograph (Fig. S9) showed no color change of liquid from the observation of separator after pre-sodiation, indicating that electrolyte was in stable state, further affirming the effectiveness of above-mentioned ameliorated techniques.

To corroborate the efficiency of Na_2_C_2_O_4_ as sacrificial additive for in situ pre-sodiation, various proportions of Na_2_C_2_O_4_ were added to AC cathode to construct full SICs (TiO_2_ as anode). The electrochemical properties of TiO_2_//AC-Na_2_C_2_O_4_-15%, TiO_2_//AC-Na_2_C_2_O_4_-20% and TiO_2_//AC-Na_2_C_2_O_4_-30% SICs have been investigated (Fig. 6c). As illustrated in Fig. 6d, note that TiO_2_//AC-Na_2_C_2_O_4_ SIC without pre-sodiation could not exhibit any available performance, with the assistance of 30 wt% of NCO-S-3D (compared to the mass of AC), it delivered an excellent energy density of 56.4 Wh kg^−1^ at power density of 20 W kg^−1^, which is almost two times as the energy density of traditional lead-acid batteries. Notably, TiO_2_//AC-NCO-S-3D-30% exhibited good rate performance, it could still manifest an energy density of 34.2 Wh kg^−1^ at power density of 2000 W kg^−1^. Meanwhile, TiO_2_//AC-Na_2_C_2_O_4_-15% and TiO_2_//AC-Na_2_C_2_O_4_-20% SICs exhibited preferable electrochemical performance (Fig. 6e–f). Nevertheless, the stabilities of TiO_2_//AC-NCO-S-3D-20% and TiO_2_//AC-NCO-S-3D-30% were not as eminent as that of TiO_2_//AC-NCO-S-3D-15% (Fig. S10), which may be caused by the depositing of Na dendritic on the surface of anode as excess sodium sources were introduced to the systems. Moreover, the mass loading of active material in cathode was around 5 mg cm^−2^, which was heavier than that of other SICs reported, implying the feasibility for large-scaled manufacture. To verify this possibility, pouch-type capacitors (AC as cathode, TiO_2_ as anode) were built with supply of 15 wt.% of sodium oxalate in cathode. Surprisingly, it delivered a preferable energy density of 40.5 Wh kg^−1^ at power density of 20 W kg^−1^ after activation according to the results in Fig. 6g. Besides, to confirm the universality of this pre-sodiation strategy, hard carbon (HC) was exploited as new anode. Based on Eq. (1) and the sodium storage capacity of HC (Fig. S11), the mass ratio of HC/AC is around 1:7.5. Impressively, the full capacitor manifested an amazing energy density of 118.2 Wh kg^−1^ at power density of 20 W kg^−1^ with only 30 wt.% of NCO-S-3D (compare to the mass of AC) (Fig. 6h–i), and outstanding rate performance was delivered (96.3 Wh kg^−1^ at 2000 W kg^−1^), indicating highly reversible trait of the electrochemical reaction even at high current rates. Additionally, admirable cycle stability was maintained after 300 cycles at the current density of 1 C as illustrated in Fig. S12. It is worth mentioning that recent work of SICs exhibited inferior energy density with much more dosage of sacrificial compound (normally 100 wt% to AC), further highlighting the superiority of our amelioration method as well as the practicability for scale-up industrialization. Inspired by the achievement of sodium oxalate in SICs, Li_2_C_2_O_4_ and K_2_C_2_O_4_ were further investigated as sacrificial cathode additives in lithium ion capacitors and potassium ion capacitors, respectively. As depicted in Fig. S13, it was found that electrochemical performance of LICs and KICs have been remarkably improved after pre-metallation compared with no additive group (no available performance). To conclude, pre-metallation triggered by the ameliorated sacrificial metal oxalate was revealed to be universal and feasible in MICs. Tremendously enhanced energy densities as well as the long cycle stabilities indicates the propelling process of commercialization for MICs.

### Mechanism Interpretation

**Fig. 7:**
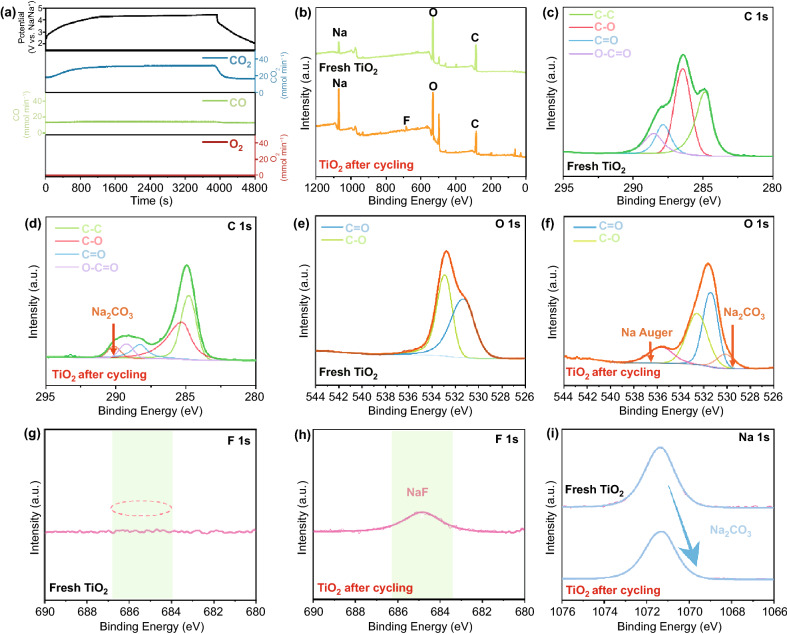
**a** Gas evolution of Na_2_C_2_O_4_ in DEMS test. **b** XPS survey spectrum TiO_2_ anode before and after cycling. C 1s high-resolution of TiO_2_ anode before (**c**) and after cycling (**d**). O 1s high-resolution of TiO_2_ anode before (**e**) and after cycling (**f**). F 1s high-resolution of TiO_2_ anode before (**g**) and after cycling (**h**). **i** Na 1s high-resolution of TiO_2_ anode before and after cycling

Importantly, thorough understanding of pre-metallation cathode additive was exploited by revealing the corresponding decomposition mechanism and the impact about formation of SEI on the anode through in situ DEMS, ex situ SEM, and ex situ XPS measurements. Typically, the similar configuration (Na_2_C_2_O_4_ as cathode, sodium metal as counter electrode) as half-cell was deployed in DEMS test. According to Fig. 7a, the gas evolution during the decomposition process of Na_2_C_2_O_4_ were monitored for the first time. Substantive release of CO_2_ was detected during the charging process, which coincides with the GCD curve, indicating the status of main product in the oxidation procedure. Meanwhile, slight CO content was examined, which may be caused by the slide reactions as no release of oxygen was traced. Ultimately, all the test results could be utilized to unveil the mask of decomposition reaction of Na_2_C_2_O_4_:

2 $${\text{Na}}_{2} {\text{C}}_{2} {\text{O}}_{4} \to 2{\text{CO}}_{2} + 2{\text{Na}}^{ + } + 2{\text{e}}^{ - }$$

Notably, benefiting from the merit of stable and innocuous product, sodium oxalate can be widely adopted without concerning about safety issues. Moreover, owing to the trait of ultra-low dosage of additive, swelling phenomenon in coin-type SICs were not appeared after cycling (Fig. S14). Besides, secondary package technique of pouch batteries could perfectly eject the gases produced during pre-sodiation procedure, thus achieving the accomplishment of no residue in the system, which ulteriorly indicates the potential to propel the implementation of Na_2_C_2_O_4_ in large-scale market. Ex situ SEM and ex situ XPS measurements have been carried out in full capacitors for further detecting the SEI film formed on TiO_2_ anode in details. As seen from SEM images (Fig. S15), the tight surface of anode was accomplished triggered by the pre-sodiation process, which could guarantee the excellent cycle performance of SICs. Moreover, the realization of pre-sodiation could be affirmed by the ex situ XPS results. The full survey scan spectrum (Fig. 7b) reveals the existence of relative elements. Na_2_CO_3_, as the vital component of SEI was appeared as new peaks in C 1s and O 1s high-resolution spectra, locating at 290.1 and 530.1 eV, respectively (Fig. 7c–f). In addition, the fresh peak at 684.3 eV detected in the F 1s spectra of cycled TiO_2_ anode ascribed to the formation of NaF, which was caused by the decomposition of fluoroethylene carbonate additive in electrolyte (Fig. 7g–h). Furthermore, Na 1s high-resolution spectrum of cycled TiO_2_ is slightly shifted to the lower binding energy site, convincingly exhibiting the formation of SEI film (Fig. 7i) [42, 43]. Such irrefutable results confirm that in situ pre-sodiation strategy could effectively replenish the sodium sources for anodes, thus boosting the electrochemical performance of SICs.

## Conclusion

In summary, with the merits of high irreversible capacity, air-stable ability, no residue and low-cost, metal oxalates (M_2_C_2_O_4_) are demonstrated as optimal sacrificial additives to accomplish the purpose of pre-metallation with sufficiently liberation of metal ions in MICs. Significantly, integrating the results of theoretical calculation and experiment, it is revealed that the strongly coupled interface strategy and electron-donating effect could be utilized to regulate decomposition potential of metal oxalates by impairing polarization phenomenon and rising HOMO levels, respectively, which are ensured by disappeared band gap as well as reduced Gibbs free energy demanded for decomposition, further addressing the issue of high activation potential. Notably, the decomposition potential of sodium oxalate can be ultimately diminished to 3.95 V through constructing interfacial bonding (Na–O–C) between sodium oxalate and 3D conductive network. Significantly, the original double coating technology mended SICs (coin-type and pouch-type) delivered excellent capacity retention with the addition of only 15 wt% of Na_2_C_2_O_4_. Besides, the potential-modified full SICs (AC//HC) could exhibit an outstanding energy density of 118.2 Wh kg^−1^ at power density of 20 W kg^−1^ as well as preeminent rate performance. More meaningfully, our strategies provide universal guidelines to address the serious overpotential hazard for cathode additives, thus promoting the wide application of ultra-low-dose sacrificial strategy. In-depth understanding about the influence of sacrificial reagent on the whole system during the electrochemical process has been further disclosed, providing theoretical guidance in terms of wielding pre-metallation techniques. Moreover, the universality and feasibility of lithium oxalate/potassium oxalate as cathode additives in LICs/KICs have been proved with dramatically enhanced performance, offering more possibilities to pave the path for the commercialization of MICs.

## Supplementary Information

Below is the link to the electronic supplementary material.

Supplementary file1 (PDF 1880 kb)
