# Supplementary material for: Ultra-Low-Dose Pre-Metallation Strategy Served for Commercial Metal-Ion Capacitors
Source: Nanomicro Lett. 2022 Jan 29;14:53. doi: 10.1007/s40820-022-00792-x (PMC8800971; doi:10.1007/s40820-022-00792-x)
Supplement: Supplementary file 1 — Supplementary file1 (PDF 1880 kb) [file 40820_2022_792_MOESM1_ESM.pdf]

Supporting Information for

Ultra-Low-Dose Pre-Metallation Strategy Served for Commercial Metal-Ion Capacitors

Zirui Song[1], Guiyu Zhang[1], Xinglan Deng[1], Kangyu Zou[1], Xuhuan Xiao[1], Roya Momen[1], Abouzar Massoudi[2], Wentao Deng[1], Jiugang Hu[1], Hongshuai Hou[1], Guoqiang Zou[1, *], and Xiaobo Ji[1, 3]

[1]College of Chemistry and Chemical Engineering, Central South University, Changsha, 410083, P. R. China

[2]Department of Semiconductors Materials and Energy Research Center P.O. Box 14155/4777, Tehran, Iran

[3]College of Material Science and Engineering, Zhengzhou University, Zhengzhou 450001, P. R. China

*Corresponding author. E-mail: gq-zou@csu.edu.cn (Guoqiang Zou)

Supplementary Figures and Tables

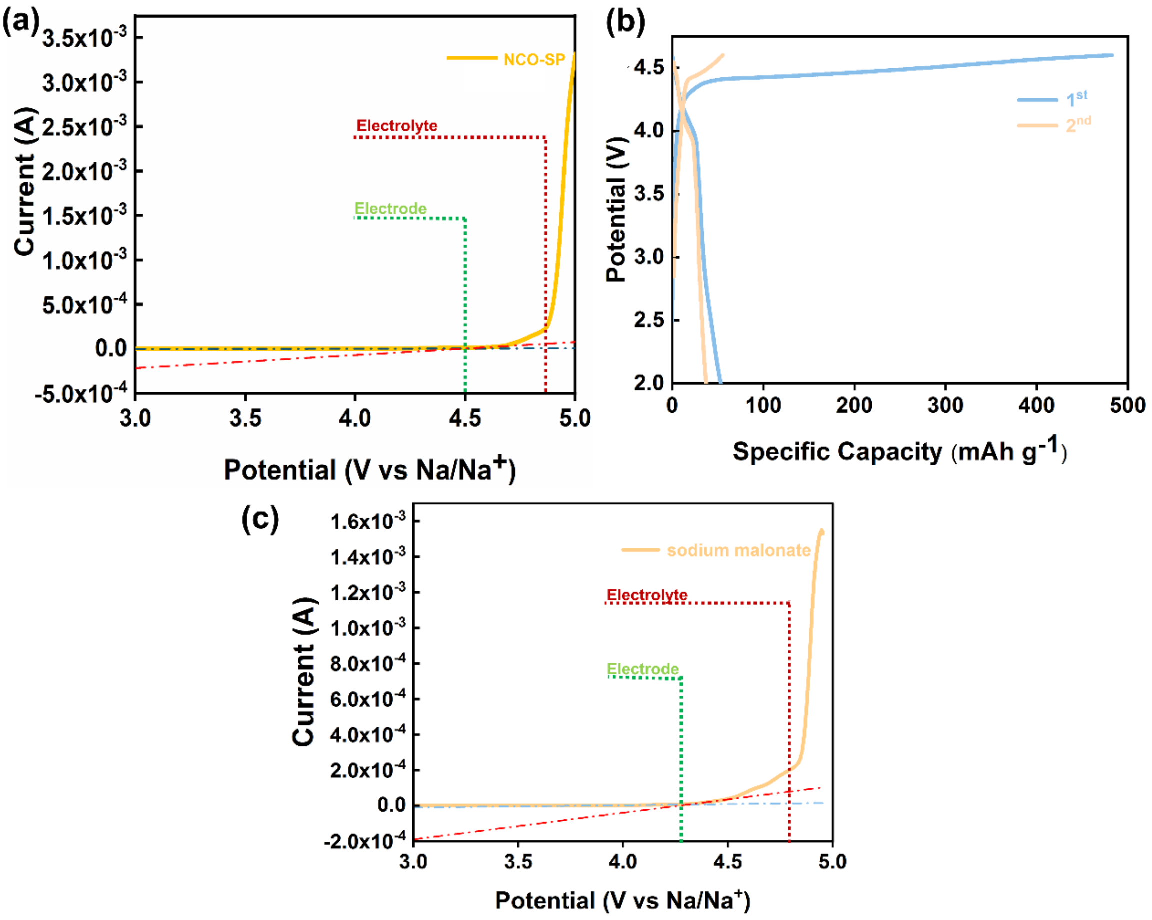

Fig. S1 Electrochemical decomposition performance of NCO-SP electrode investigated by (**a**) CV curves at 0.25 mV s^{-1}. (**b**) GCD curves of NCO-SP at the current density of 0.1C within the potential range of 4.6-2 V. (**c**) CV curves of $Na_2H_2C_3O_4$-SP at 0.25 mV s^{-1}

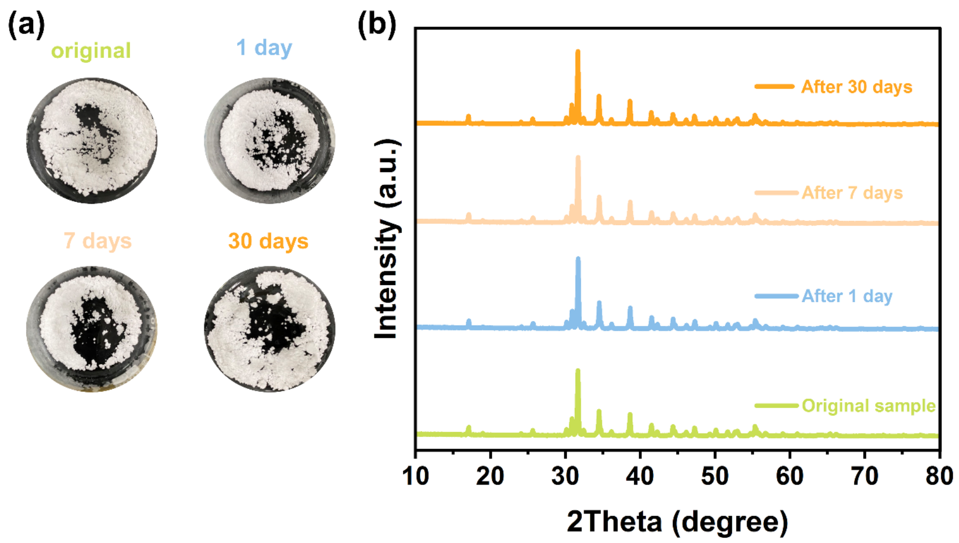

Fig. S2 Status of sodium oxalate after being exposed in ambient environment for certain days. (**a**) Optical photos. (**b**) XRD patterns

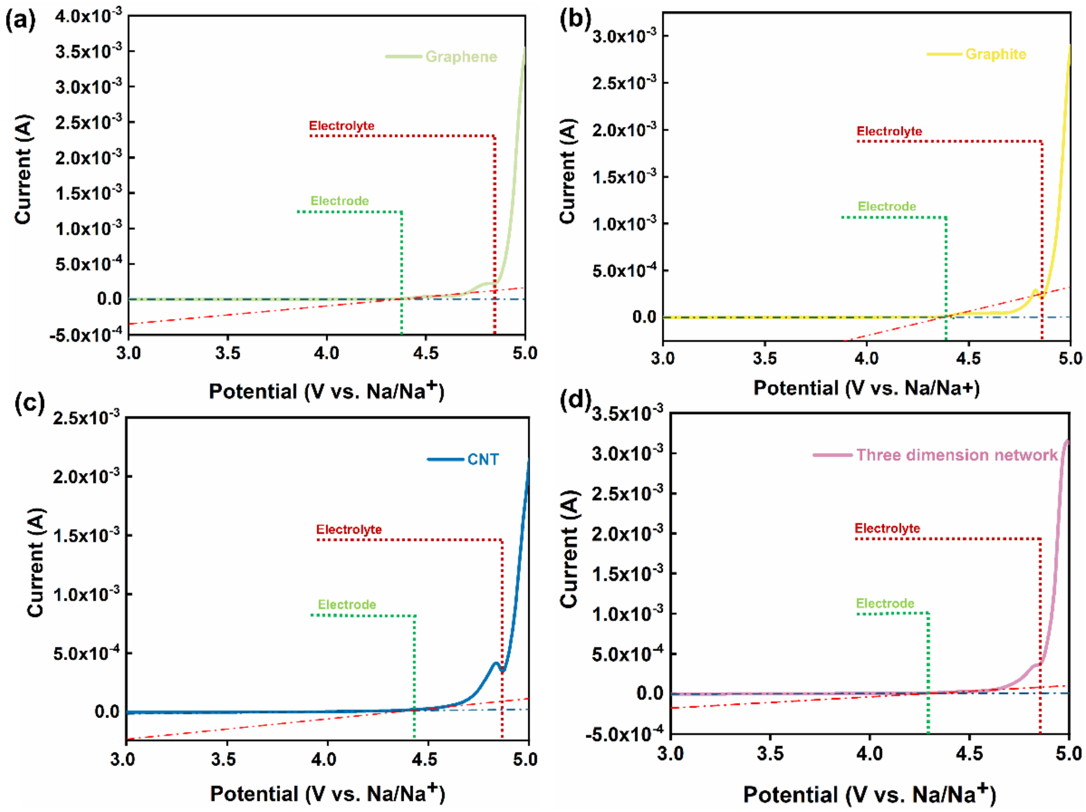

Fig. S3 CV curves of $Na_2C_2O_4$ in various conductive additive systems at 0.25 mV s^{-1}. (**a**) graphene system. (**b**) graphite system. (**c**) carbon nanotube system. (**d**) 3D conductive network system

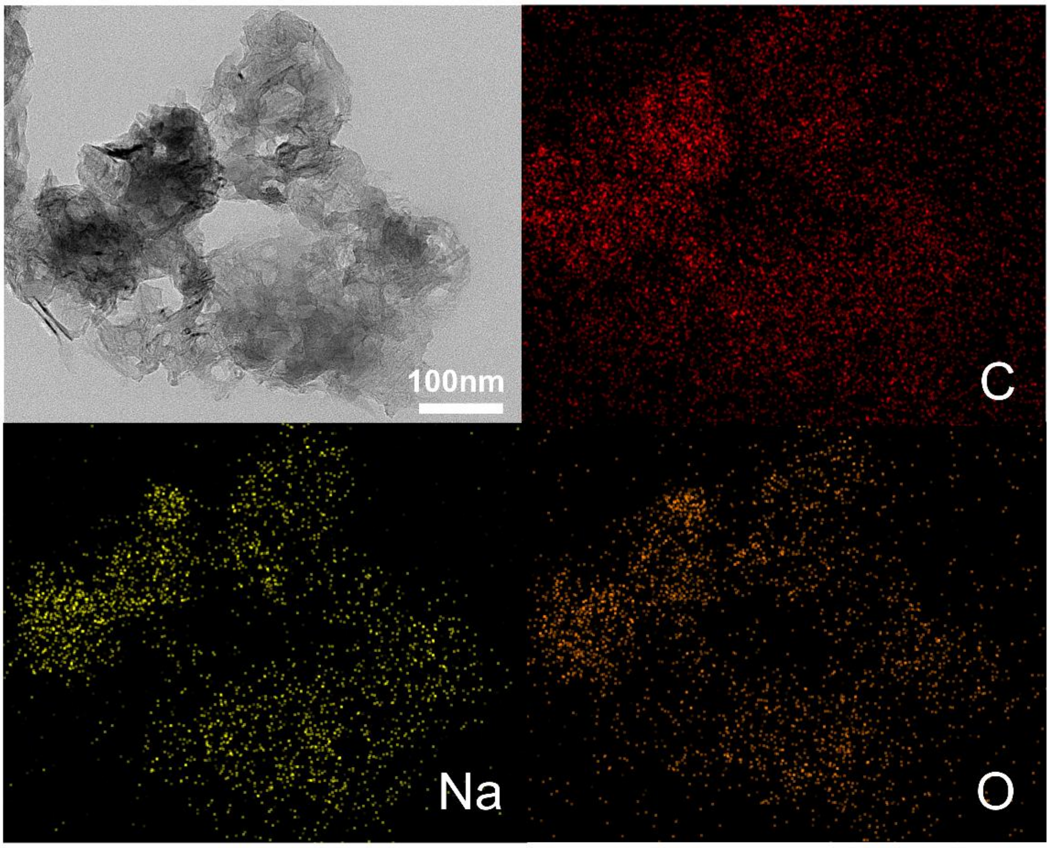

Fig. S4 TEM image of NCO-S-3D and corresponding EDS elemental mapping

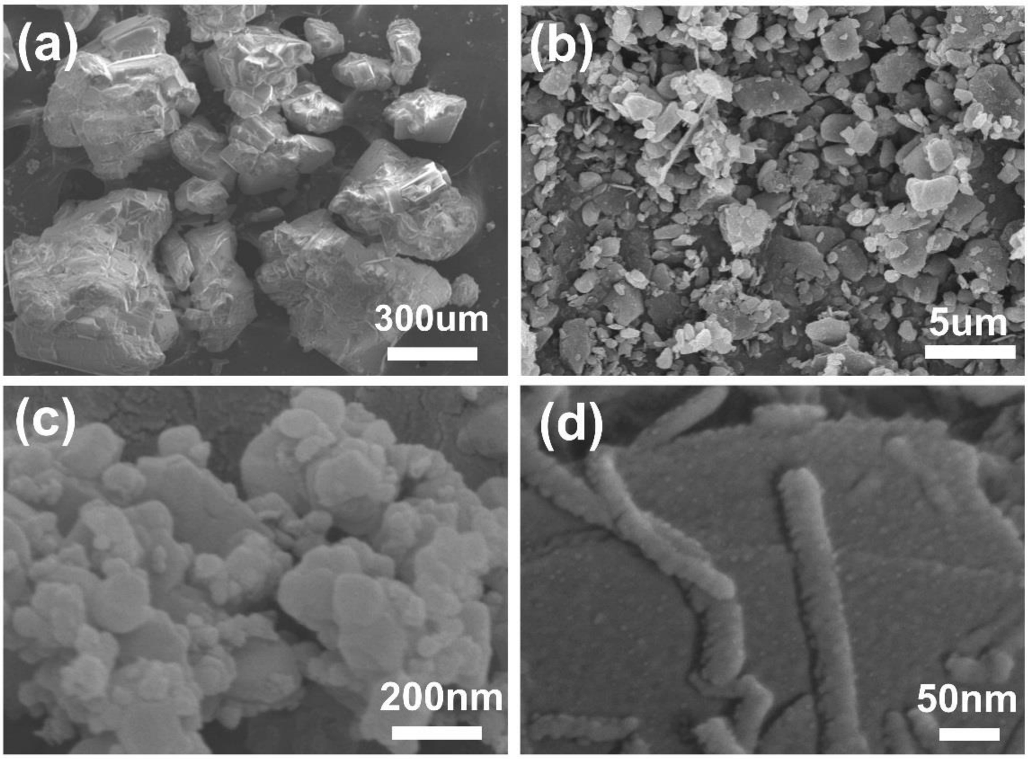

Fig. S5 SEM images of (**a**) pristine sodium oxalate and (**b-d**) after being ball milled with 3D conductive network

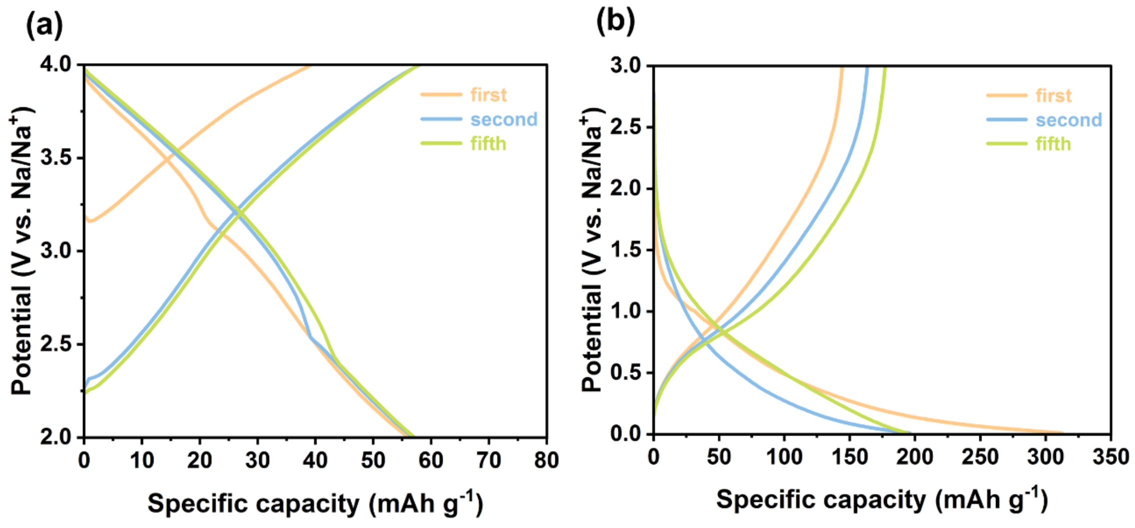

Fig. S6 Charge and discharge profiles of (**a**) activated carbon and (**b**) TiO_2 at the current density of 100 mA g^{-1}

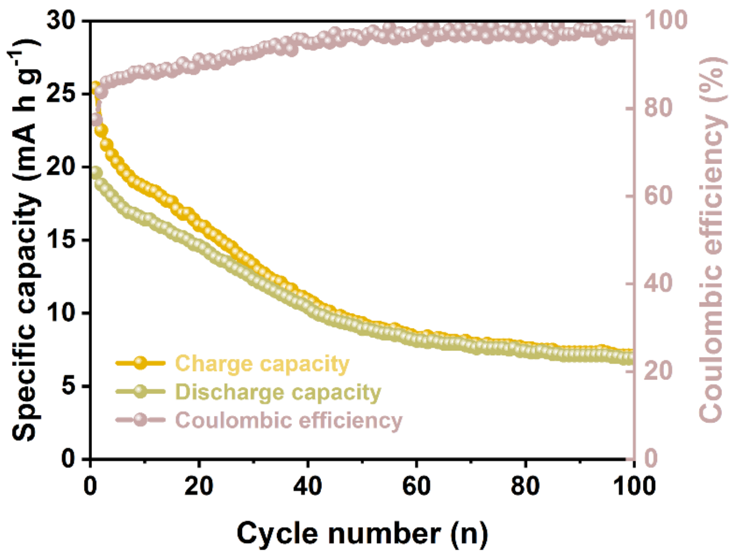

Fig. S7 Cycling performance and CE of TiO_2//AC-NCO-15% with traditional slurry method under 1C at 4-0 V after presodiation

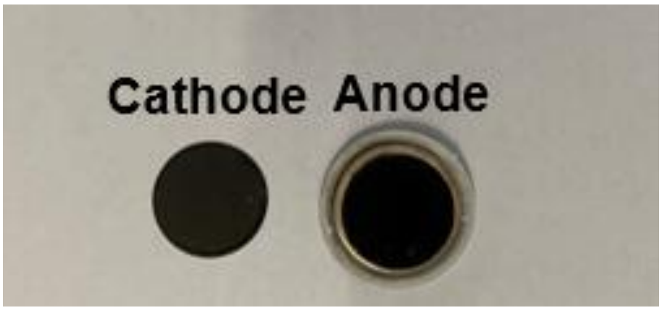

Fig. S8 Optical photos of electrodes in disassembled SIC system after cycling for 100 cycles

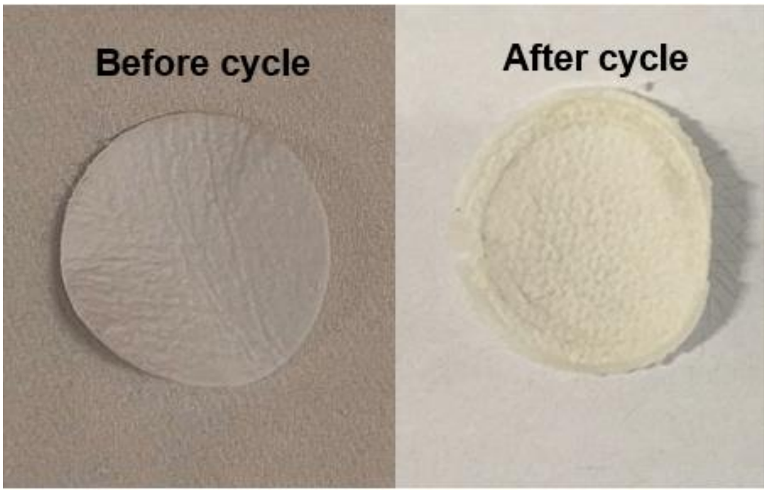

Fig. S9 Optical photos of separators disassembled SIC system after cycling for 100 cycles

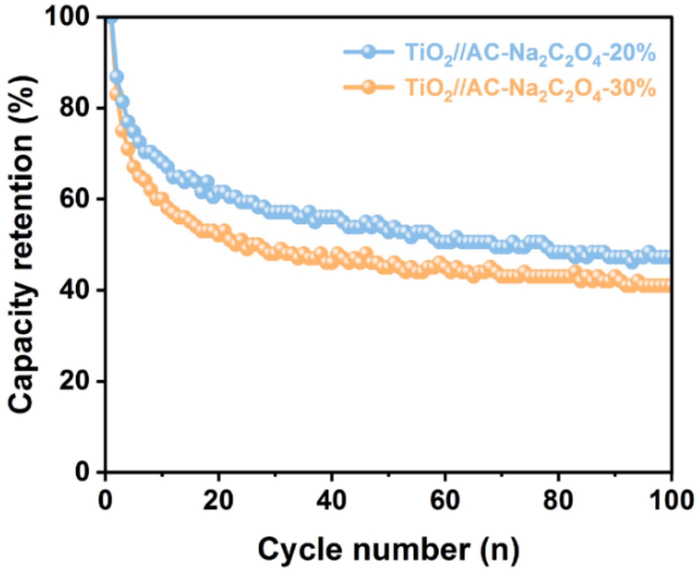

Fig. S10 Cycling stability of TiO_2//AC-NCO-S-3D-20% and TiO_2//AC-NCO-S-3D-30% SICs at 1C within 4-0 V after presodiation

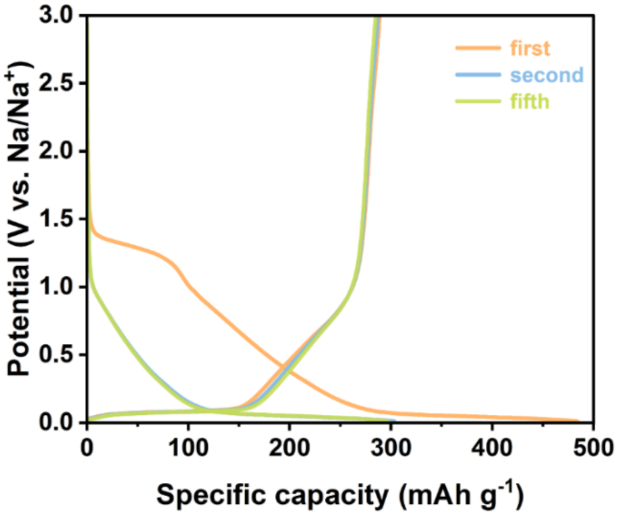

Fig. S11 Charge and discharge profiles of hard carbon at the current density of 100 mA g^{-1}

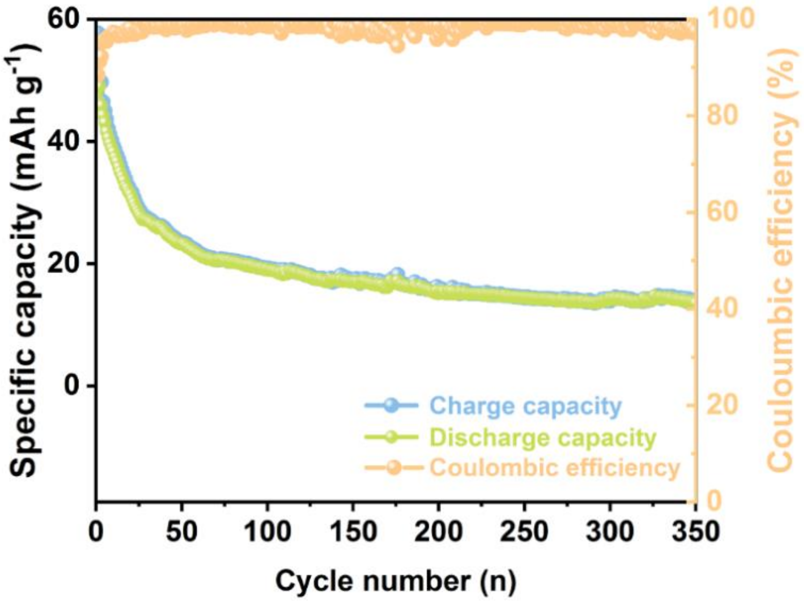

Fig. S12 Cycling performance and CE of HC//AC-NCO-30% under 1C at 4-0 V after presodiation

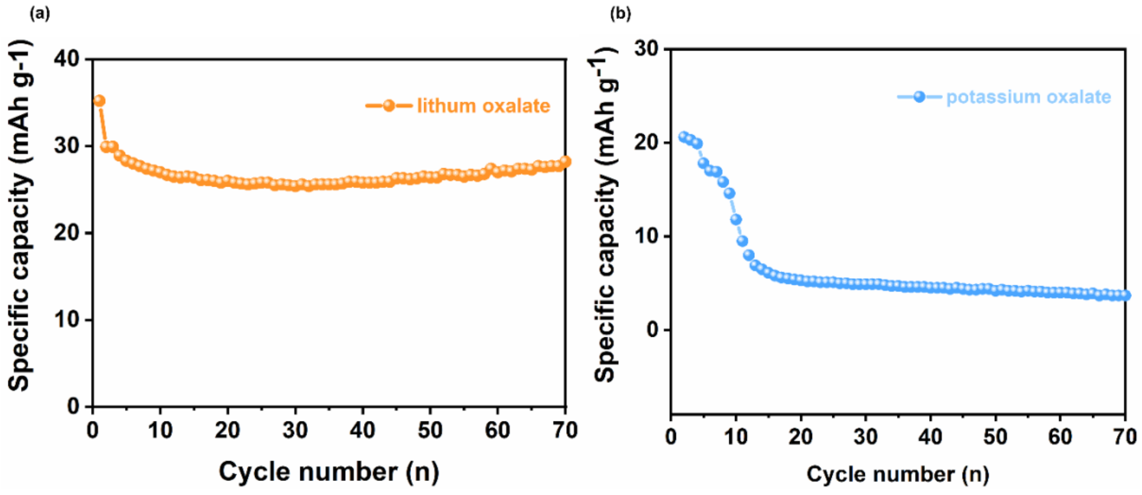

Fig. S13 Cycling performance of (**a**) TiO_2//AC-$Li_2C_2O_4$-15% and (**b**) TiO_2//AC-$K_2C_2O_4$-15% under 1C at 4-0 V after premetallation

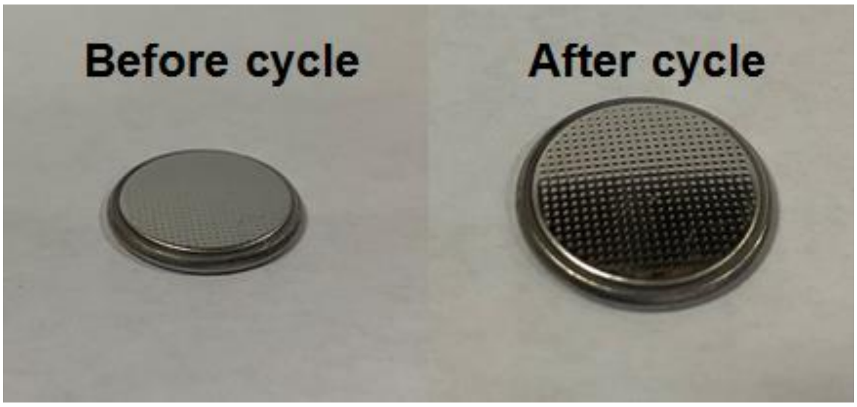

Fig. S14 Observation of coin-type SIC before and after cycling

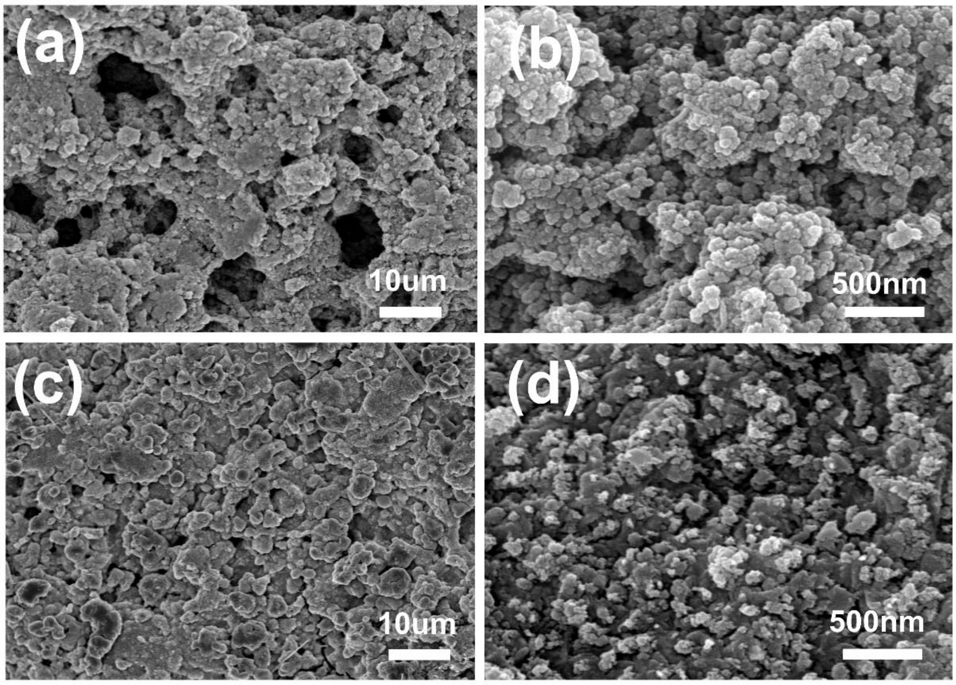

Fig. S15 Surface images of TiO_2 anode before (**a, b**) and after (**c, d**) cycles

Table S1 Properties of sacrificial cathode additives currently applied in sodium ion capacitors

Sacrificial additives	Decomposition product	Theoretical capacity (mA h g^{-1})	Dosage (compared to active cathode)	Cost (CNY kg^{-1})	Purity
**Na_2S**	S	687	100%	50000	95%
**$Na_2C_4O_4$**	CO_2/C	339	80%	Lab-synthesis	Lab-synthesis
**$Na_2C_4O_4$**	CO_2/CO/C	339	58%/220%	Lab-synthesis	Lab-synthesis
**$Na_2C_6O_6$**	C_6O_6	250	60%/100%/167%	4400	Analytical Reagent
**$NaNH_2$**	$NH_2NH_2/N_2/H_2$	686	45%	328	95%
**$Na_2C_2O_4$ (our work)**	CO_2	400	15%/20%/30%	5	99.8%

Table S2 Summary of decomposition voltages of sacrificial cathode additives related to various amelioration methods

	NCO-SP	$Na_2H_2C_3O_4$-SP	NCO-graphene	NCO-graphite	NCO-carbon nanotube	NCO-3D	NCO-S-3D
**Decomposition potential (V)**	4.50	4.30	4.36	4.38	4.40	4.30	**3.95**

Abbreviations:

NCO: $Na_2C_2O_4$; 3D: Three dimension conductivenetwork;

NCO-SP: $Na_2C_2O_4$ with super p; NCO-S-3D: size-reduced $Na_2C_2O_4$ with 3D network.
